# Genome-wide DNA methylation and long-term ambient air pollution exposure in Korean adults

**DOI:** 10.1186/s13148-019-0635-z

**Published:** 2019-02-28

**Authors:** Mi Kyeong Lee, Cheng-Jian Xu, Megan U. Carnes, Cody E. Nichols, James M. Ward, The BIOS consortium, Sung Ok Kwon, Sun-Young Kim, Woo Jin Kim, Stephanie J. London

**Affiliations:** 1 Epidemiology Branch, Division of Intramural Research, Department of Health and Human Services, National Institute of Environmental Health Sciences, National Institutes of Health, Research Triangle Park, NC 27709 USA; 2 Department of Pediatric Pulmonology and Pediatric Allergy, Beatrix Children’s Hospital, University of Groningen, University Medical Center Groningen, Groningen, The Netherlands; 3 Department of Genetics, University of Groningen, University Medical Center Groningen, Groningen, The Netherlands; 4 GRIAC Research Institute, University of Groningen, University Medical Center Groningen, Groningen, The Netherlands; 5 OmicSoft, QIAGEN Bioinformatics, Cary, NC 27513 USA; 6 Department of Internal Medicine and Environmental Health Center, Kangwon National University Hospital, School of Medicine, Kangwon National University, Chuncheon, 24289 South Korea; 7 Department of Cancer Control and Population Health, Graduate School of Cancer Science and Policy, National Cancer Center, Goyang, 10408 South Korea

**Keywords:** Air pollution, Particulate matter, Nitrogen dioxide, Epigenesis, genetic, Epigenomics

## Abstract

**Background:**

Ambient air pollution is associated with numerous adverse health outcomes, but the underlying mechanisms are not well understood; epigenetic effects including altered DNA methylation could play a role. To evaluate associations of long-term air pollution exposure with DNA methylation in blood, we conducted an epigenome-wide association study in a Korean chronic obstructive pulmonary disease cohort (*N* = 100 including 60 cases) using Illumina’s Infinium HumanMethylation450K Beadchip. Annual average concentrations of particulate matter ≤ 10 μm in diameter (PM_10_) and nitrogen dioxide (NO_2_) were estimated at participants’ residential addresses using exposure prediction models. We used robust linear regression to identify differentially methylated probes (DMPs) and two different approaches, DMRcate and comb-p, to identify differentially methylated regions (DMRs).

**Results:**

After multiple testing correction (false discovery rate < 0.05), there were 12 DMPs and 27 DMRs associated with PM_10_ and 45 DMPs and 57 DMRs related to NO_2_. DMP cg06992688 (*OTUB2*) and several DMRs were associated with both exposures. Eleven DMPs in relation to NO_2_ confirmed previous findings in Europeans; the remainder were novel. Methylation levels of 39 DMPs were associated with expression levels of nearby genes in a separate dataset of 3075 individuals. Enriched networks were related to outcomes associated with air pollution including cardiovascular and respiratory diseases as well as inflammatory and immune responses.

**Conclusions:**

This study provides evidence that long-term ambient air pollution exposure impacts DNA methylation. The differential methylation signals can serve as potential air pollution biomarkers. These results may help better understand the influences of ambient air pollution on human health.

**Electronic supplementary material:**

The online version of this article (10.1186/s13148-019-0635-z) contains supplementary material, which is available to authorized users.

## Background

Exposure to ambient air pollution has well-documented adverse effects on health outcomes, including cardiovascular disease [1] and pulmonary function [2]. Oxidative stress and inflammation have been suggested as underlying mechanisms but specific data supporting these links are lacking. Despite mounting evidence of the negative impacts of air pollution exposure on health outcomes, the underlying mechanisms are not well understood.

DNA methylation, an epigenetic modification that can influence gene expression, has widely replicated genome-wide associations with smoking [3]. While there are fewer data, there is evidence that ambient air pollution influences methylation [4–7]. Most studies of long-term air pollution exposure and methylation have been conducted in Caucasian adult populations [5–7] and evidence for replication of differentially methylated probes (DMPs) across studies or different ethnic groups is sparse.

We performed an epigenome-wide association study (EWAS) to evaluate the relationship of long-term exposure to particulate matter ≤ 10 μm in diameter (PM_10_) and nitrogen dioxide (NO_2_) with blood DNA methylation in adults (*N* = 100) participating in a Korean chronic obstructive pulmonary disease (COPD) cohort. We identified differentially methylated signals in relation to air pollution exposure both at an individual C–phosphate–G (CpG) probe level and at a regional level involving several neighboring CpG probes (CpGs). We evaluated whether methylation levels of our DMPs were associated with expression levels of nearby transcripts in a large independent dataset with matched gene expression and DNA methylation in the same individuals, Biobank-based integrative omics studies (BIOS) consortium. We also replicated findings from earlier EWASes in European populations, reporting a list of DMPs showing similar associations in our Asian population.

## Methods

### Study population

For DNA methylation profiling, study participants (*N* = 100 including 60 individuals with COPD) were sampled from a Korean COPD cohort [8]. Data and biologic specimens collected at a baseline visit (between late August and early November in 2012 and 2013) were used in this study. Blood and urine samples as well as survey questionnaires were obtained for all study participants who also underwent physical examination for anthropometric measurements. A trained nurse measured height and weight using the body composition analyzer IOI 353 (Aarna Systems., Udaipur, India). Body mass index (BMI) was calculated as weight (kg) divided by height squared (m^2^). Information on cigarette smoking status (never, former, and current) and pack-years of smoking was obtained via questionnaires. We calculated pack-years of smoking, for current and former smokers, by multiplying the number of years smoked by the number of cigarette packs smoked per day. Current nonsmoking status was validated using urine cotinine levels (nmol/L) measured by immunoassay (Immulite 2000 Xpi; Siemens Healthcare Diagnostics, Tarrytown, NY, USA). Workflow of this study can be found in Additional file 1: Figure S1. The study protocol was approved by the Institutional Review Board at Kangwon National University. We obtained informed consent from all study participants.

### Air pollution exposure at residential addresses

We estimated annual average concentrations of PM_10_ (μg/m^3^) and NO_2_ (ppb) at each residential address obtained from the baseline survey using a national-scale exposure prediction model [9]. Using air pollution regulatory monitoring data in 2010, the prediction model estimated the annual average concentrations of the pollutants in a universal kriging framework based on geographic predictors and spatial correlation. Geographic predictors were estimated by hundreds of geographic variables that represent pollution sources including traffic, demographic characteristics, land use, physical geography, transportation facilities, emissions, vegetation, and altitude. To account for season in the prediction model, we used several inclusion criteria for monitoring sites: (1) having more than 75% (274 days) of daily data, (2) having at least one daily measurement in each of the 10 months, and (3) having no more than 45 consecutive days without daily measurements. Participants’ residential addresses at the baseline visit were geocoded using GeoCoder-Xr software (Geoservice, Seoul, South Korea).

### DNA methylation profiling

DNA was extracted from blood samples collected at the baseline visit. We obtained genome-wide methylation profiles using the Infinium HumanMethylation450K BeadChip (Illumina, Inc., San Diego, CA, USA). We used a pipeline implemented in the chip analysis methylation pipeline (ChAMP) R package [10] for signal extraction and initial low-quality probe filtering, excluding probes having a detection *p* value > 0.01 in any sample or a bead-count < 3 in 5% or more samples. Correction for probe design bias was done using Beta Mixture Quantile dilation normalization [11]. Batch effects were corrected using Combat [12] in the sva R package [13]. To minimize false positive findings, we additionally removed non-CpG probes and probes reported to be non-specific [14, 15] or potentially influenced by nearby single-nucleotide variants [14]. We provide probe filtering steps in Additional file 2: Table S1. After excluding probes on the X and Y chromosomes, the remaining 402,508 CpGs were used for association analyses. To reduce the potential influence of extreme methylation outliers on association results, we removed methylation values more extreme than Tukey’s outer fences [16] defined as more than three times the interquartile range from the 25th and 75th percentiles of methylation values at each probe, resulting in removal of 75,549 (0.19%) values across all participants. To estimate cell-type proportions including CD8^+^ T lymphocytes, CD4^+^ T lymphocytes, natural killer cells, B cells, monocytes, and granulocytes, we applied Houseman’s algorithm [17] with the Reinius reference panel [18] using the minfi R package [19].

### Identification of differentially methylated probes

To evaluate associations of air pollution exposure with DNA methylation, we used robust linear regression models to decrease the influence of outlier methylation values and heteroskedasticity on association results [20]. Annual average concentrations of a pollutant (PM_10_ or NO_2_) were used as the predictor and the methylation beta values were the response variable. A methylation beta value is a ratio of methylated CpG probe intensity to total probe intensity and ranges between 0 (unmethylated) and 1 (methylated). Covariates included were age (years), sex (male, female), cigarette smoking (never, former, current), pack-years of smoking, BMI (kg/m^2^), COPD status (cases, noncases), and estimated cell-type proportions. For genome-wide statistical significance, we set a threshold of Benjamini-Hochberg false discovery rate (FDR) adjusted *p* value < 0.05 unless otherwise noted. We also used *p* value < 1.2E-07 (= 0.05/402,508) as a cutoff for statistically significant associations after Bonferroni correction. We used R version 3.0.2 for preprocessing methylation data from raw data (.idat files) to methylation beta values and R version 3.4.0 for association analyses and visualization of differential methylation regions.

### Identification of differentially methylated regions

In addition to association analyses at individual CpGs, we applied two different methods to identify differential DNA methylation at the regional level in relation to air pollution exposure: DMRcate [21] and comb-p [22]. As the two methods implement different algorithms to identify differentially methylated regions (DMRs), we used both methods to find significant DMRs while reducing false positives. DMRcate uses a tunable kernel smoothing process with differential methylation association signals, whereas comb-p examines regional clustering of low *p* values from irregularly spaced *p* values. We used the “dmrcate” function in the DMRcate R package with input files from the epigenome-wide association results: regression coefficients, standard deviations, and uncorrected *p* values. Comb-p, a stand-alone software, was used with input files containing uncorrected *p* values and information on chromosomal locations (chromosome and physical position). To define significant DMRs in our study, we applied the following three criteria. First, more than one CpG should reside within a DMR. Second, regional differential methylation signals can be calculated using neighboring CpGs within 1000 base pairs (bp). Third, a region must have multiple-testing corrected *p* value < 0.05 in both methods: Benjamini-Hochberg FDR for DMRcate and Sidak for comb-p. The use of FDR for DMRcate and Sidak for comb-p was the default setting in the two methods. As the minimum number of CpGs (*N* = 2) in a region and the minimum length of a distance (*N* = 1000 nucleotides) were the defaults in DMRcate, we used the same values for comb-p to harmonize results from the two methods. As the two methods call DMRs based on association results of neighboring probes, a significant DMR does not necessarily overlap a significant differentially methylated probe (DMP) in that region (Additional file 2: Table S2 and S3). To visualize regions of differential methylation, we used the coMET R package [23].

### Biological implications of association results

Gene annotation for each CpG was done by using the manufacturer’s annotation file [24]; the UCSC RefGene names were obtained. For biological implications of our differential methylation signals in relation to each pollutant (PM_10_ or NO_2_), we explored curated variant annotations in the GeneticsLand software (OmicSoft, QIAGEN, NC, USA) and performed functional pathway analyses using the “Core Analysis” of ingenuity pathway analysis (IPA; Ingenuity Systems, QIAGEN, CA, USA) on genes annotated to DMPs with an uncorrected *p* value < 1E-04 (an arbitrary cutoff for suggestive association) or significant DMRs. To assess enrichment of tissue- or cell type-specific signals, we analyzed DMPs (FDR < 0.05) and probes having the minimum *p* value in each DMR for overlap with DNase 1 hypersensitivity sites (DHSs) using the experimentally derived functional element overlap analysis of ReGions from EWAS (eFORGE, version 1.2) [25].

### Replication look-up

To replicate our DMPs with results from previous EWASes, we looked for evidence of our DMPs (FDR < 0.05) in the two published epigenome-wide studies of PM_10_ and/or NO_2_ exposure in adults [6, 7]. Also, we examined whether DMPs reported in the two studies were replicated in our study. Across the two studies, 5001 DMPs were reported (FDR < 0.05): 9 for PM_10_ and 4992 for NO_2_. Of these, 4671 were available for the look-up analysis in our data after probe filtering: 9 for PM_10_ and 4662 for NO_2_. We set the cutoff of an uncorrected *p* value < 0.05 for statistical significance for the look-up.

### Associations of methylation levels of DMPs with gene expression levels of nearby transcripts: expression quantitative trait methylation in the BIOS data

To evaluate associations between methylation levels of DMPs and expression levels of nearby transcripts (cis-eQTMs), we regressed the methylation *M* value, the log2 ratio of methylated versus unmethylated probe intensities, on gene expression, adjusting for age, sex, lymphocytes percentage, monocyte percentage, and RNA flow cell number. The inflation of models was corrected using the “bacon” method [26]. We mapped the expression quantitative trait methylation (eQTMs) in a window of 250 kilobase pairs (kb) around the significant DMPs (FDR < 0.05). For this analysis, we used a total of 3075 samples for which both methylation and gene expression data were available from 4 cohorts: Leiden Longevity Study, LifeLines Study, Rotterdam Study, and Netherland Twin Study. We analyzed each cohort separately and then meta-analyzed the results using the inverse variance-weighted fixed-effects model using METAL software [27].

## Results

The average age of the study participants was 73 years (standard deviation, SD = 6) and 66% were male (Table 1). There were 39 never, 30 former, and 31 current smokers. The mean annual average concentration was 45.1 μg/m^3^ for PM_10_ and 13.1 ppb for NO_2_. The two air pollutants were highly correlated (Spearman correlation coefficient = 0.74, *p* value < 2.2E-16).

**Table 1:** Descriptive characteristics of the study population

Characteristics	The Korean COPD^a^ cohort (*N* = 100)
Age, years	72.8 ± 6.3
Male	66 (66%)
Body mass index, kg/m^2^	22.9 ± 2.9
COPD, case	60 (60%)
Cigarette smoking	
Never	39 (39%)
Former	30 (30%)
Current	31 (31%)
Pack-years	
Former smoker	28.9 ± 19.6
Current smoker	35.7 ± 19.1
Annual average air pollution concentration at residential addresses	
PM_10_, μg/m^3^	45.1 ± 2.0
NO_2_, ppb	13.1 ± 1.4

*N* (%) or mean ± standard deviation reported

^a^Chronic obstructive pulmonary disease

We observed numerous DMPs in relation to the two pollutants (FDR < 0.05): 11 for PM_10_ alone, 44 for NO_2_ alone, and 1 for both PM_10_ and NO_2_ (Tables 2 and 3). Of these 56 DMPs, some showed statistical significance after Bonferroni multiple testing correction: cg05454562 (*WDR46*), cg13999433 (*AKNA*), and cg11691844 (*SYTL2*) associated with PM_10_ exposure (Table 2); cg05171937 (*STK38L*), cg26583725 (8541 bp apart from *IRS2*), and cg06226567 (*C20orf56*) associated with NO_2_ exposure (Table 3). The DMP cg06992688 (*OTUB2*) was positively associated with both PM_10_ and NO_2_ (FDR < 0.05). Exposure to the two pollutants was mostly positively associated with DNA methylation: 92% (*N* = 11/12 CpGs) for PM_10_ and 71% (*N* = 32/45 CpGs) for NO_2_. In Additional file 1: Figure S2, we provide Manhattan and quantile-quantile plots for visual representation of the epigenome-wide association results (Additional file 3). No systematic inflation was observed in our results as genomic inflation factor (lambda) values were 0.83 for PM_10_ exposure and 1.07 for NO_2_ exposure.

**Table 2:** Differentially methylated CpGs in blood DNA in relation to PM_10_ exposure (FDR < 0.05), ordered by chromosomal location

Chr^a^	Gene (distance to gene^b^)	Probe	Position^c^	Coef^d^	SE^e^	P^f^
1	*NEGR1*	cg07721244	72749275	0.004	0.001	1.6E-07
2	*ARID5A*	cg04722215	97205147	− 0.006	0.001	1.4E-07
3	*FOXL2* (− 81,364)	cg21742790	138581702	0.005	0.001	8.6E-07
3	*XXYLT1* (− 92,147)	cg04252203	194696866	0.005	0.001	6.7E-07
6	*WDR46*	cg05454562^g^	33254447	0.006	0.001	4.3E-09
7	*FAM20C* (− 5283)	cg16998831	187686	0.008	0.002	3.0E-07
8	*KIF13B*	cg07023317	28961315	0.008	0.002	1.4E-06
9	*AKNA*	cg13999433^g^	117156883	0.007	0.001	3.9E-08
11	*SYTL2*	cg11691844^g^	85460604	0.006	0.001	1.1E-07
14	*OTUB2*	cg06992688	94491958	0.008	0.002	1.0E-06
16	*MIR5093* (11,6079)	cg26964426	85455911	0.025	0.005	8.3E-07
18	*NPC1*	cg12709880	21163172	0.007	0.001	3.8E-07

^a^Chromosome

^b^Distance to transcription start site of the mapped gene (base pair)

^c^Physical position (base pair, National Center for Biotechnology Information human reference genome assembly Build 37.3)

^d^Regression coefficient from statistical model. Covariates age, sex, cigarette smoking status, pack-years of smoking, BMI, COPD status, and estimated cell-type proportions were included in the model. The coefficient can be interpreted as the difference in DNA methylation per 1 μg/m^3^ PM_10_ exposure. For example, cg07721244 showed 0.4% methylation increase per 1 μg/m^3^ PM_10_ exposure increase. Methylation values range 0–1

^e^Standard error of regression coefficient

^f^Uncorrected *p* value

^g^Statistically significant after Bonferroni multiple-testing correction (1.2E-07)

**Table 3:** Differentially methylated CpGs in blood DNA in relation to NO_2_ exposure (FDR < 0.05), ordered by chromosomal location

Chr^a^	Gene (distance to gene^b^)	Probe	Position^c^	Coef^d^	SE^e^	P^f^
1	*MAN1C1* (− 7282)	cg16396978	25936677	0.008	0.002	3.9E-06
1	*ERI3*	cg13451048	44820073	0.007	0.001	8.6E-07
1	*RPL5*	cg02769668	93302925	− 0.003	0.001	3.3E-07
1	*WARS2* (− 29,067)	cg06764239	119544772	0.002	3.5E-04	4.3E-06
1	*S100A12*	cg02901136	153348305	0.012	0.002	2.7E-06
2	*STON1* (169)	cg23256664	48757477	− 0.001	3.0E-04	8.9E-07
2	*NDUFB3*	cg04865026	201936505	0.012	0.002	7.5E-07
2	*PLEKHM3*	cg09950920	208734940	0.013	0.003	2.7E-07
2	*PIKFYVE*	cg19351166	209133632	0.008	0.002	5.5E-06
3	*CTDSPL*	cg12386061	37906586	0.002	4.3E-04	5.5E-06
3	*DCBLD2* (122,596)	cg01188562	98637410	− 0.004	0.001	2.0E-06
3	*AP2M1*	cg17343451	183899704	0.009	0.002	3.3E-06
4	*CPLX1*	cg16649791	816968	− 0.014	0.003	1.0E-06
4	*LINC01097* (− 3902)	cg25913520	13524041	0.002	3.6E-04	2.8E-06
4	*LOC641518*	cg13775316	109093218	0.002	4.0E-04	6.2E-07
5	*DAP* (86217)	cg23112301	10765559	− 0.005	0.001	3.8E-06
5	*ZNF366*	cg21770462	71803219	0.008	0.002	4.7E-06
5	*ERAP1* (− 82,414)	cg13625213	95915327	− 0.002	4.0E-04	3.4E-06
5	*CDHR2* (− 2294)	cg18194153	175967218	0.010	0.002	1.3E-07
6	*LTA*	cg11586857	31540136	− 0.007	0.001	3.5E-06
8	*PMP2*	cg22796481	82353365	− 0.019	0.004	6.7E-07
8	*OSR2*	cg09607488	99963657	0.007	0.002	4.5E-06
9	*RORB*	cg04130427	77113915	0.005	0.001	3.7E-06
10	*ZNF438*	cg10575075	31288634	0.014	0.003	2.0E-06
10	*EMX2*	cg02420850	119302157	0.002	4.0E-04	6.2E-07
11	*TMEM138*	cg03370752	61136373	0.010	0.002	5.5E-06
11	*SORL1*	cg17510957	121466629	0.011	0.002	5.1E-06
12	*TEAD4*	cg12902426	3068889	0.003	0.001	3.7E-06
12	*STK38L*	cg05171937^g^	27396765	0.010	0.002	1.1E-08
12	*DDX55*	cg13559144	124086193	0.002	4.3E-04	3.0E-06
13	*EDNRB*	cg23326536	78491199	− 0.003	0.001	1.7E-06
13	*IRS2* (−8541)	cg26583725^g^	110397643	− 0.001	2.3E-04	4.9E-08
14	*ITPK1*	cg05284742	93552128	0.009	0.002	4.1E-06
14	*OTUB2*	cg06992688	94491958	0.013	0.003	3.3E-06
14	*PLD4*	cg15352829	105391018	0.010	0.002	3.0E-06
15	*LOC145663*	cg04025675	45671028	0.005	0.001	6.3E-07
16	*ZCCHC14*	cg16727006	87470545	− 0.010	0.002	4.8E-06
17	*EFCAB5* (− 2689)	cg22888787	27950276	0.010	0.002	3.9E-07
17	*CD300A* (− 12,486)	cg00227781	72450036	0.004	0.001	3.0E-06
19	*LOC100128675*	cg06642503	35597415	− 0.005	0.001	2.9E-06
19	*ZNF347*	cg15050103	53642858	− 0.008	0.002	3.7E-06
19	*ZNF542* (− 28,810)	cg06109293	56850658	0.020	0.004	1.9E-07
20	*NKX2-4* (− 3198)	cg27650906	21372807	0.006	0.001	3.1E-07
20	*C20orf56*	cg06226567^g^	22559676	0.003	0.001	3.5E-08
21	*MORC3*	cg01261013	37691747	0.010	0.002	4.1E-06

^a^Chromosome

^b^Distance to transcription start site of the mapped gene (base pair)

^c^Physical position (base pair, National Center for Biotechnology Information human reference genome assembly Build 37.3)

^d^Regression coefficient from statistical model. Covariates age, sex, cigarette smoking status, pack-years of smoking, BMI, COPD status and estimated cell-type proportions were included in the model. The coefficient can be interpreted as the difference in DNA methylation per 1 ppb NO_2_ exposure. For example, cg16396978 showed 0.8% methylation increase per 1 ppb NO_2_ exposure increase. Methylation values range 0–1

^e^Standard error of regression coefficient

^f^Uncorrected *p* value

^g^Statistically significant after Bonferroni multiple-testing correction (1.2E-07)

We found numerous DMRs in relation to air pollution exposure: 22 for PM_10_ alone, 52 for NO_2_ alone, and 5 for both PM_10_ and NO_2_ (Tables 4 and 5). The five DMRs associated with both pollutants were chr6:30297174-30297627 (*TRIM39*), chr6:31539539-31540750 (*LTA*), chr8:19459672-19460243 (*CSGALNACT1*), chr17:80084554-80085082 (*CCDC57*), and chr20:45179157-45179413 (*C20orf123*).

**Table 4:** Differentially methylated regions in blood DNA in relation to PM_10_ exposure (adjusted *P* < 0.05 both in DMRcate and in comb-p)

Chr^a^	Gene (distance to gene^b^)	DMRcate				comb-p				Minimum P^g^
		Start (bp^c^)	End (bp)	FDR^d^	#CpGs^e^	Start (bp)	End (bp)	Sidak P^f^	#CpGs	
1	*MIB2*	1549615	1550031	0.020	5 (4)			0.009		0.009
2	*NOL10* (−22,166)	10687583	10688726	9.4E-05	8 (5)	10687962	10688317	2.6E-05	5 (5)	2.5E-04
2	*SNED1*	241975035	241976244	0.006	6 (5)	241975756		0.015	4 (4)	0.005
3	*IL20RB*	136676672	136676846	0.007	2 (2)			0.011		2.5E-04
6	*TRIM27*	28874479	28875370	9.4E-05	7 (6)		28874754	7.3E-04	4 (4)	0.002
6	*TRIM39*	30297174	30297627	2.3E-08	11 (10)			1.1E-07		8.4E-04
6	*LTA*	31539539	31540750	1.3E-11	19 (13)		31540461	3.4E-06	18 (12)	4.8E-05
6	*TREM1*	41254471	41254997	0.018	4 (3)	41254433		0.012	5 (3)	1.7E-04
7	*FOXK1*	4752951	4753002	1.3E-04	3 (3)			7.2E-04		3.4E-04
8	*CSGALNACT1*	19459672	19460243	0.003	7 (4)			0.001		7.8E-04
8	*PIWIL2*	22131675	22133356	1.2E-04	15 (6)	22132563		0.027	13 (5)	3.8E-05
8	*KIF13B* ^h^	28961315	28961356	2.9E-04	3 (2)			0.003		1.4E-06
9	*C9orf131*	35042344	35042395	0.003	2 (2)			0.005		5.6E-05
10	*CAMK1D*	12648032	12648338	3.6E-02	3 (2)		12648526	0.011	4 (3)	0.002
10	*C10orf105*	73498624	73498766	0.003	3 (2)			0.032		2.7E-05
10	*PTPRE*	129794994	129795003	0.002	2 (2)			0.020		3.9E-05
15	*FLJ42289*	100890907	100891257	1.1E-04	5 (4)	100890963		0.014	4 (3)	8.8E-05
17	*TNRC6C*	76036514	76037562	7.3E-05	7 (7)	76037035		1.6E-05	6 (6)	0.001
17	*CCDC57*	80084554	80085082	1.3E-04	4 (4)			1.3E-05		4.3E-05
19	*PRTN3*	846117	846354	0.010	3 (3)			0.004		0.001
19	*PRTN3*	847943	848071	0.005	4 (4)			0.003		0.001
19	*CALR*	13053719	13054718	0.002	5 (4)	13054427		0.014	4 (3)	8.3E-05
19	*FBXO17*	39465821	39466757	0.002	8 (4)			0.004		6.6E-04
20	*STK35*	2085157	2085344	0.003	2 (2)			0.002		1.7E-05
20	*SLPI*	43882990	43883307	0.004	3 (3)		43883546	8.5E-04	4 (4)	9.7E-04
20	*C20orf123*	45179157	45179413	2.0E-04	6 (5)			1.4E-04		2.2E-04
21	*C21orf81*	15352848	15352983	0.013	2 (2)			0.012		4.7E-04

Blanked cells in “Start,” “End,” and “#CpGs” for comb-p represent the same information compared to results in DMRcate

^a^Chromosome

^b^Minimum distance to transcription start site of the mapped gene (base pair)

^c^Physical position (base pair, National Center for Biotechnology Information human reference genome assembly Build 37.3)

^d^Benjamini-Hochberg false discovery rate

^e^Number of probes in the region (number of probes having uncorrected *p* value < 0.05)

^f^P of Sidak multiple-testing correction

^g^Minimum *p* value among uncorrected p-values of CpGs in each region. When either start or end positions were different between DMRs from the two DMR approaches, we used results from DMRcate

^h^Region including significant (FDR < 0.05) differentially methylated probes from our epigenome-wide association study

**Table 5:** Differentially methylated regions in blood DNA in relation to NO_2_ exposure (adjusted *p* value < 0.05 both in DMRcate and in comb-p)

Chr^a^	Gene (distance to gene^b^)	DMRcate				comb-p				Minimum P^g^
		Start (bp^c^)	End (bp)	FDR^d^	#CpGs^e^	Start (bp)	End (bp)	Sidak P^f^	#CpGs	
1	*RUNX3*	25291041	25291905	0.005	7 (4)		25291584	0.044	6 (3)	0.001
1	*RPS6KA1*	26855423	26855926	0.006	4 (3)		26855765	0.009	3 (3)	9.1E-04
1	*TFAP2E*	36038468	36039173	2.1E-04	8 (7)	36038701		3.3E-04	6 (6)	0.002
1	*ARTN*	44398868	44399894	5.6E-04	10 (6)	44399363		0.012	6 (4)	9.8E-04
1	*S100A12* ^h^	153347819	153348305	2.8E-04	2 (2)			0.005		2.7E-06
1	*S100A14*	153589528	153590243	0.013	4 (2)	153589781		0.047	3 (2)	0.001
1	*S100A13*	153599479	153600156	0.001	7 (6)			6.6E-04		0.003
1	*ATP8B2*	154300117	154300241	6.9E-04	2 (2)			0.007		3.4E-05
1	*LAX1*	203733971	203734559	0.004	6 (4)			0.002		0.002
1	*C1orf35*	228291118	228291705	0.023	6 (5)			0.017		0.009
2	*ALS2CR11*	202483704	202484583	0.007	10 (5)	202484020		0.008	7 (5)	0.006
3	*AMT*	49459143	49460521	1.3E-06	10 (7)	49459855		8.3E-05	9 (7)	1.6E-04
3	*PPM1L*	160475035	160475336	0.002	5 (5)			0.003		0.002
3	*B3GALNT1*	160822268	160822911	0.001	8 (5)		160822711	0.031	5 (4)	0.003
5	*MGAT4B*	179230709	179231109	0.002	3 (2)			0.006		2.5E-04
5	*OR2V1* (− 39,287)	180511822	180512070	0.012	2 (2)			0.020		7.8E-04
6	*DUSP22*	291687	292823	7.6E-04	9 (8)	291882		2.2E-04	8 (7)	0.005
6	*TRIM39*	30297174	30297627	1.5E-06	11 (9)			2.9E-05		6.7E-04
6	*LTA* ^h^	31539539	31540750	1.9E-15	19 (11)		31540461	4.5E-07	18 (11)	3.5E-06
6	*HLA-DMB*	32904074	32905190	1.2E-05	9 (5)		32904621	0.001	5 (3)	8.7E-06
6	*HLA-DPB2*	33083989	33085470	2.5E-06	22 (12)	33084420		2.3E-04	21 (11)	0.006
6	*TRAF3IP2*	111887243	111887834	0.002	2 (2)			0.026		3.2E-04
6	*MLLT4*; *C6orf124*	168227843	168228706	0.001	3 (3)	168228374		0.004	2 (2)	6.4E-05
7	*UNCX* (− 5426)	1266180	1267228	8.2E-04	4 (4)	1266616		0.001	3 (3)	2.0E-04
7	*EVX1* (− 2589)	27279101	27279575	0.009	3 (2)			0.044		1.8E-04
7	*STEAP2*	89840396	89841435	1.9E-05	13 (5)		89841214	0.004	12 (5)	2.1E-04
8	*CSGALNACT1*	19459672	19460243	2.3E-05	7 (5)			6.7E-05		1.4E-04
8	*KIAA0146*; *CEBPD* (−19)	48648112	48649767	7.6E-08	7 (7)	48648813		3.9E-09	6 (6)	7.4E-05
8	*HEY1*	80678770	80679314	0.002	4 (3)		80678925	0.026	2 (2)	4.4E-04
8	*NDRG1*	134307105	134307728	2.3E-05	3 (3)	134307597		7.4E-04	2 (2)	3.0E-05
10	*HK1*	71087924	71088038	0.009	2 (2)			0.038		2.0E-04
10	*LRRC20*	72141375	72141924	7.0E-06	5 (3)		72141625	0.007	4 (3)	1.2E-05
10	*HTRA1*	124254773	124254860	0.003	2 (2)			0.010		1.1E-04
11	*IFITM3* (10863)	330536	331179	5.1E-04	5 (3)			0.001		1.2E-05
11	*LMO2*	33913187	33914088	9.3E-04	5 (4)			3.2E-04		7.2E-04
11	*ME3* (− 9563)	86142104	86142587	5.1E-04	4 (3)			0.012		1.0E-04
13	*PDX1* (− 1903)	28491326	28492265	0.006	8 (3)	28491409	28491975	0.035	6 (3)	0.001
13	*PCDH20*	61989203	61990025	6.0E-05	12 (8)	61989701		5.3E-04	8 (7)	6.7E-04
13	*DAOA* (−319,060)	105791890	105792346	0.023	3 (3)			0.024		0.003
14	*DAD1* (−58,286)	22974144	22975521	0.007	6 (5)	22974951		0.029	5 (4)	1.2E-04
14	*CTSG*	25045625	25046121	0.013	3 (3)			0.008		0.002
14	*PLD4* ^h^	105390602	105391263	0.002	3 (2)	105391018		0.007	2 (2)	3.0E-06
15	*GATM*; *LOC145663*^h^	45670068	45671708	8.3E-08	17 (7)	45670478	45671347	1.2E-04	14 (7)	6.4E-07
15	*TNFAIP8L3*	51387571	51387921	0.002	5 (5)			0.004		9.7E-04
15	*FLJ42289*	100890441	100891257	8.3E-07	9 (4)	100890907		1.0E-05	5 (4)	3.3E-05
16	*TMEM8A*; *LOC100134368*	432973	434356	1.3E-05	7 (4)	433439	433825	1.1E-04	5 (4)	1.2E-04
16	*CLDN9*	3062056	3062975	0.001	8 (6)	3062349		8.0E-04	7 (6)	0.005
17	*ALOX12*	6898738	6900356	6.9E-10	16 (12)		6899888	1.9E-08	15 (12)	0.001
17	*WNK4*	40932199	40932983	0.006	11 (6)		40932746	0.005	9 (6)	0.011
17	*IGF2BP1*	47091521	47092272	0.006	6 (5)	47091978		0.042	5 (4)	0.003
17	*CCDC57*	80084554	80085082	0.003	4 (4)			0.001		0.002
19	*ELANE*	855536	856107	4.5E-04	4 (4)			6.1E-05		2.8E-04
19	*FBXO17*	39465821	39467258	2.0E-04	9 (6)		39466757	6.1E-05	8 (6)	0.003
20	*C20orf123*	45179157	45179413	0.002	6 (5)			0.005		0.002
21	*RUNX1*	36259067	36259797	0.005	5 (4)		36259460	0.008	4 (4)	0.003
22	*PARVG*	44568337	44568812	0.024	9 (5)			0.043		0.006
22	*PRR5*	45125218	45126040	0.002	5 (4)		45125666	0.005	4 (3)	0.002

Blanked cells in “Start,” “End,” and “#CpGs” for comb-p represent the same information compared to results in DMRcate

^a^Chromosome

^b^Minimum distance to transcription start site of the mapped gene (base pair)

^c^Physical position (base pair, National Center for Biotechnology Information human reference genome assembly Build 37.3)

^d^Benjamini-Hochberg false discovery rate

^e^Number of probes in the region (number of probes having uncorrected *p* value < 0.05)

^f^P of Sidak multiple-testing correction

^g^Minimum *p* value among uncorrected p-values of CpGs in the region. When either start or end positions were different between DMRs from the two DMR approaches, we used results from DMRcate

^h^Region including significant (FDR < 0.05) differentially methylated probes from our epigenome-wide association study

Although a DMR does not necessarily contain a DMP, one DMR related to PM_10_ exposure chr8:28961315-28961356 (*KIF13B*) contains a DMP—cg07023317. Four DMRs associated with NO_2_ exposure contain a DMP: cg02901136 in chr1:153347819-153348305 (*S100A12*), cg11586857 in chr6:31539539-31540750 (*LTA*), cg15352829 in chr14:105390602-105391263 (*PLD4*), and cg04025675 in chr15:45670068-45671708 (*GATM*; LOC145663). From each DMR method, the top two DMRs based on multiple-testing corrected *p* values (FDR from DMRcate) were visualized for regional association results including annotation of regulatory genomic regions and pairwise correlation of neighboring probes (Additional file 1: Figure S3).

We identified biological networks enriched in our association results based on genes to which either DMPs (FDR < 0.05) or CpGs having the minimum *p* value within the DMRs (FDR < 0.05 in DMRcate, Sidak adjusted *p* value < 0.05 in comb-p) were annotated: 138 for PM_10_ and 288 for NO_2_. The enriched networks included inflammatory and immune responses and cardiovascular, respiratory, and metabolic diseases (Additional file 2: Table S4 and S5). Cancer, hematological development, immunological and inflammatory diseases pathways overlap between PM_10_ and NO_2_ related differential methylation signals (Additional file 1: Figure S4. A). Of the genes associated with both PM_10_ and NO_2_ exposure, several contribute to the hematological, immunological, and inflammatory networks: *NLRC4*, *RPTOR*, *CUX1*, *S100A12*, *LTA*, and *HLA-DMB* (Additional file 1: Figure S4. B).

Using eFORGE [25], we found some enriched tissue- or cell type-specific histone marks (H3K27me3, H3K36me3, H3K4me3, H3K9me3, and H3K4me1) among the 132 probes associated with air pollution (PM_10_ or NO_2_) exposure based on either FDR < 0.05 from the DMP analyses or the minimum *p* value in the DMRs: 11 DMPs for PM_10_ exposure alone, 44 DMPs for NO_2_ exposure alone, 1 DMP for both PM_10_ and NO_2_ exposure, 19 probes showing the minimum *p* value in PM_10_ exposure related DMRs, 49 probes showing the minimum *p* value in NO_2_ exposure related DMRs, and 8 probes showing the minimum *p* value in DMRs associated with both PM_10_ and NO_2_ exposure. Enrichment of H3K4me1 in blood was observed for differential methylation related to PM_10_ exposure (Additional file 1: Figure S5). With respect to differential methylation related to NO_2_ exposure, several histone marks were enriched: H3K4me1, H3K27me3, H3K4me3, and H3K9me3 in blood; H3K4me1 and H3K27me3 in embryonic stem (ES) cell; and H3K4me1 in lung (Additional file 1: Figure S6).

Several DMPs (FDR < 0.05) in our study were reported to be associated with air pollution exposure in previous genome-wide DNA methylation studies. Of the 27 DMPs associated with NO_2_ (FDR < 0.05) in our study, 11 were reported to be related to NO_2_ exposure with the same direction of effects (Table 6) in the LifeLines cohort [7]. The 12 DMPs related to PM_10_ (FDR < 0.05) in our study were novel, meaning not reported to be associated with this pollutant in either of the two earlier studies [6, 7]. Notably, of the 4662 probes reported to be associated with NO_2_ exposure in the 2 studies and also available in our data, 26% (*N* = 1231) showed associations in our study of at least nominal significance (uncorrected *p* value < 0.05) with the same direction of effects (Additional file 2: Table S6).

**Table 6:** Look-up analysis of CpGs associated with NO_2_ exposure in the Korean COPD Cohort (FDR < 0.05) in a previous publication from the LifeLines Cohort from the Netherlands

Chr^a^	Gene (distance to gene^b^)	Probe	The Korean COPD study		The LifeLines cohort study [7]	
			Coef^c^ (per 1 ppb NO_2_) ± SE^d^	*P* ^e^	Coef (per 10 μg/m^3^ NO_2_) ± SE	*P*
1	*MAN1C1* (− 7282)	cg16396978	0.008 ± 0.002	3.9E-06	0.013 ± 0.004	5.4E-04
1	*S100A12*	cg02901136	0.012 ± 0.002	2.7E-06	0.027 ± 0.006	3.1E-05
2	*PLEKHM3*	cg09950920	0.013 ± 0.003	2.7E-07	0.024 ± 0.007	3.4E-04
3	*AP2M1*	cg17343451	0.009 ± 0.002	3.3E-06	0.020 ± 0.005	1.4E-05
5	*ZNF366*	cg21770462	0.008 ± 0.002	4.7E-06	0.015 ± 0.004	4.1E-05
10	*ZNF438*	cg10575075	0.014 ± 0.003	2.0E-06	0.026 ± 0.007	2.7E-04
11	*TMEM138*	cg03370752	0.010 ± 0.002	5.5E-06	0.028 ± 0.008	2.4E-04
11	*SORL1*	cg17510957	0.011 ± 0.002	5.1E-06	0.023 ± 0.007	4.7E-04
12	*STK38L*	cg05171937	0.010 ± 0.002	1.1E-08	0.036 ± 0.009	4.0E-05
14	*OTUB2*	cg06992688	0.013 ± 0.003	3.3E-06	0.026 ± 0.007	1.4E-04
21	*MORC3*	cg01261013	0.010 ± 0.002	4.1E-06	0.023 ± 0.006	3.3E-04

^a^Chromosome

^b^Distance to transcription start site of the mapped gene (base pair)

^c^Regression coefficient from statistical model

^d^Standard error of regression coefficient

^e^Statistical significance from statistical model

From the analyses linking DNA methylation and gene expression in the BIOS data, we observed correlations of methylation levels of DMPs with gene expression levels of nearby (spanning a 250 bp window) transcripts (uncorrected *p* value < 0.05). Notably, of the 56 DMPs (FDR < 0.05), 70% (*N* = 39) were significantly related to gene expression of nearby transcripts (Additional file 2: Table S7).

## Discussion

To our knowledge, this is the first study of genome-wide DNA methylation in relation to long-term ambient air pollution exposure, both PM_10_ and NO_2_, in an Asian population. We identified many differentially methylated signals—both individual probes and regions—related to long-term air pollution exposure in blood. We also replicated, in our Asian population, findings from earlier studies in European populations. Of our genome-wide significant findings, some provide the first replication of an earlier report from a European population [7] while others are novel. Notably, methylation levels of many DMPs were associated with gene expression levels of nearby transcripts, providing a link between ambient air pollution exposure-related differential methylation and gene expression.

Some of our DMPs annotated to genetic loci reported in published genome-wide association studies of various health outcomes that have been related to air pollution exposure. Differential methylation of cg11586857 related to both pollutants annotated to *LTA* in which an earlier study identified rs1799964 (*p* value = 3.3E-07) to be associated with blood lipid levels [28]. Cg06992688 associated with exposure to both air pollutants resides in *OTUB2*, a nearby gene of three genetic variants related to lung function with *p* values around 1.0E-04 [29]. In addition, cg05284742 related to NO_2_ exposure is located in *ITPK1*; this gene contains rs2295394 (*p* value = 2.3E-16) associated with myocardial infarction in Asian populations [30].

Knowledge-based pathway analyses and enrichment analyses of epigenetic elements using publicly available data provided biological implication of our study findings. Enrichment of networks, such as inflammatory and immune responses and cardiovascular, pulmonary and metabolic diseases, in our results supports previous findings of air pollution exposure and the identified disease associations. Several enriched histone marks in relevant tissue and cell types (embryonic stem cell, blood and lung) suggest additional biological relevance of our differential methylation signals.

We found five studies examining associations of DNA methylation, measured using Illumina’s Infinium 450K array, with ambient air pollution exposure in either children or adults [5–7, 31, 32]. Of the five, one reported DMPs associated with short-term exposure to particulate matter < 2.5 μm (PM_2.5_) [31]. Chi and colleagues [5] measured DNA methylation using the 450K array but they analyzed only a subset of probes for associations with PM_2.5_ and oxides of nitrogen (NOx). Gruzieva and colleagues [32] found differential methylation in children in relation to prenatal NO_2_ exposure. The remaining two analyzed long-term exposure to pollutants including both PM_10_ and NO_2_ for associations with genome-wide DNA methylation in adults [6, 7]. Notably, differential methylation signals in our study provide the first replication of findings from the two studies in European adults [6, 7], suggesting similar relationships between ambient air pollution exposure and DNA methylation between European and Asian populations.

In this study, we adjusted for COPD status because it may confound associations between air pollution exposure and methylation. We also explored possible effect measure modification by the disease status in a sensitivity analysis. Of the 45 CpGs related to NO_2_, three (cg16649791, cg13559144, and cg23326536), showed an interaction term that was nominally significant (Additional file 2: Table S8); none of the 12 PM_10_-related CpGs showed statistically significant interaction.

Our study has limitations and strengths. Limitations include the lack of a replication population. However, we were able to compare our findings against published lists of DMPs at genome-wide significance from two earlier studies in European populations [6, 7]. With respect to the exposure assessment, we used exposure values at residential addresses estimated from a national-scale prediction model rather than an area-specific model which could not be developed because of the limited number of monitoring sites (< 10) in the areas where our study participants resided. However, in previous US studies, estimates of PM_2.5_ for specific areas using national models showed association results comparable to those from area-specific models [33, 34]. Third, we used annual average concentrations estimated for 2010 and participant addresses at baseline visits in 2012 without incorporating participants’ previous exposure to air pollution. The year 2010 was used in the model because of the increased number of available monitoring sites and temporally aligned geographic data. As spatial distribution of air pollution should be relatively consistent over years in our study area with stable environments, the impact of using temporally limited exposure and address information on our methylation analysis could be small. Lastly, we have a relatively small sample size compared to earlier genome-wide methylation studies of air pollution exposure.

The study has a number of important strengths. Participants reported residing in the same residential areas for 50 years (SD = 21) on average. This high level of residential stability improved our ability to estimate associations with long-term air pollution exposure. Further, we have included both PM_10_ and NO_2_ exposure so that we can examine whether there are common or unique differential methylation signals related to the two pollutants. In addition, we followed up our DMPs by examining relationships with gene expression and found that a majority were related to gene expression, suggesting functional importance of the associations. Further, we conducted pathway analyses and enrichment analyses of tissue- and cell-type specific histone marks to better understand the biological implication of the differentially methylated signals that we observed. Last, we identified DMRs by combining association signals at neighboring CpGs using two different methods in addition to identifying DMPs.

## Conclusions

We identified differential DNA methylation signals in blood associated with long-term ambient air pollution exposure and linked differential methylation to differential gene expression. Replication of many of our results from an Asian population, in a European population, suggests similar influences of air pollution exposure across ancestry. Our CpGs and regions showing differential methylation are potential biomarkers for long-term ambient air pollution exposure. These findings may better inform mechanisms linking air pollution exposure to adverse health outcomes.

## Additional files

Additional file 1: **Figure S1.** Workflow of the epigenome-wide association study of long-term ambient air pollution exposure. **Figure S2.** Manhattan and quantile-quantile plots. **Figure S3.** Regional visualization of the association of air pollution exposure (PM_10_ and NO_2_) with blood DNA methylation. **Figure S4.** Visualization of pathway analysis results. **Figure S5.** Tissue- and cell-type specific enrichment pattern in CpGs significantly associated (FDR < 0.05) with PM_10_ exposure. **Figure S6.** Tissue- and cell-type specific enrichment pattern in CpGs significantly associated (FDR < 0.05) with NO_2_ exposure (DOCX 6165 kb)

Additional file 2: **Table S1.** CpG probe filtering criteria in the 450 K array. **Table S2.** CpGs included in the top five differentially methylated regions in relation to PM_10_ from each analysis: DMRcate and comb-p (ordered by software and chromosomal location). **Table S3.** CpGs included in the top five differentially methylated regions in relation to NO_2_ from each analysis: DMRcate and comb-p (ordered by software and chromosomal location). **Table S4.** Enriched networks in genes related to PM_10_ exposure. **Table S5.** Enriched networks in genes related to NO_2_ exposure. **Table S6.** Look-up analysis of CpGs associated with NO_2_ exposure (FDR < 0.05 in earlier epigenome-wide association studies) in the Korean COPD cohort, sorted by uncorrected P in the Korean COPD Cohort. **Table S7.** Associations between methylation levels at air pollution associated CpGs (FDR < 0.05) and the expression levels of nearby genes: cis-eQTMs. **Table S8.** Differential methylation of an interaction between NO_2_ exposure and COPD status (XLSX 115 kb)

Additional file 3: **Table S9.** Differential methylation in relation to PM_10_ exposure. **Table S10.** Differential methylation in relation to NO_2_ exposure (XLSX 46770 kb)

## Abbreviations

BIOS: Biobank-based integrative omics studies
BMI: Body mass index
ChAMP: Chip analysis methylation pipeline
COPD: Chronic obstructive pulmonary disease
CpGs: C–phosphate–G probes
DMPs: Differentially methylated probes
DMRs: Differentially methylated regions
eFORGE: Experimentally-derived functional element overlap analysis of regions from EWAS
eQTM: Expression quantitative trait methylation
EWAS: Epigenome-wide association study
FDR: False discovery rate
IPA: Ingenuity pathway analysis
NO_2_: Nitrogen dioxide
PM10: Particulate matter ≤ 10 μm in diameter
SD: Standard deviation
