# Supplementary material for: Genome-wide DNA methylation and long-term ambient air pollution exposure in Korean adults
Source: Clin Epigenetics. 2019 Feb 28;11:37. doi: 10.1186/s13148-019-0635-z (PMC6396524; doi:10.1186/s13148-019-0635-z)
Supplement: Supplementary file 1 — Figure S1. Workflow of the epigenome-wide association study of long-term ambient air pollution exposure. Figure S2. Manhattan and quantile-quantile plots. Figure S3. Regional visualization of the association of air pollution exposure (PM10 and NO2) with blood DNA methylation. Figure S4. Visualization of pathway analysis results. Figure S5. Tissue- and cell-type specific enrichment pattern in CpGs significantly associated (FDR < 0.05) with PM10 exposure. Figure S6. Tissue- and cell-type specific enrichment pattern in CpGs significantly associated (FDR < 0.05) with NO2 exposure (DOCX 6165 kb) [file 13148_2019_635_MOESM1_ESM.docx]

**Supplementary Material**

Figure S1. Workflow of the epigenome-wide association study of long-term ambient air pollution exposure

Figure S2. Manhattan and quantile-quantile plots

Figure S3. Regional visualization of the association of air pollution exposure (PM_10_ and NO_2_) with blood DNA methylation

Figure S4. Visualization of pathway analysis results

Figure S5. Tissue- and cell-type specific enrichment pattern in CpGs significantly associated (FDR<0.05) with PM_10_ exposure

Figure S6. Tissue- and cell-type specific enrichment pattern in CpGs significantly associated (FDR<0.05) with NO_2_ exposure

Figure S1. Workflow of the epigenome-wide association study of long-term ambient air pollution exposure
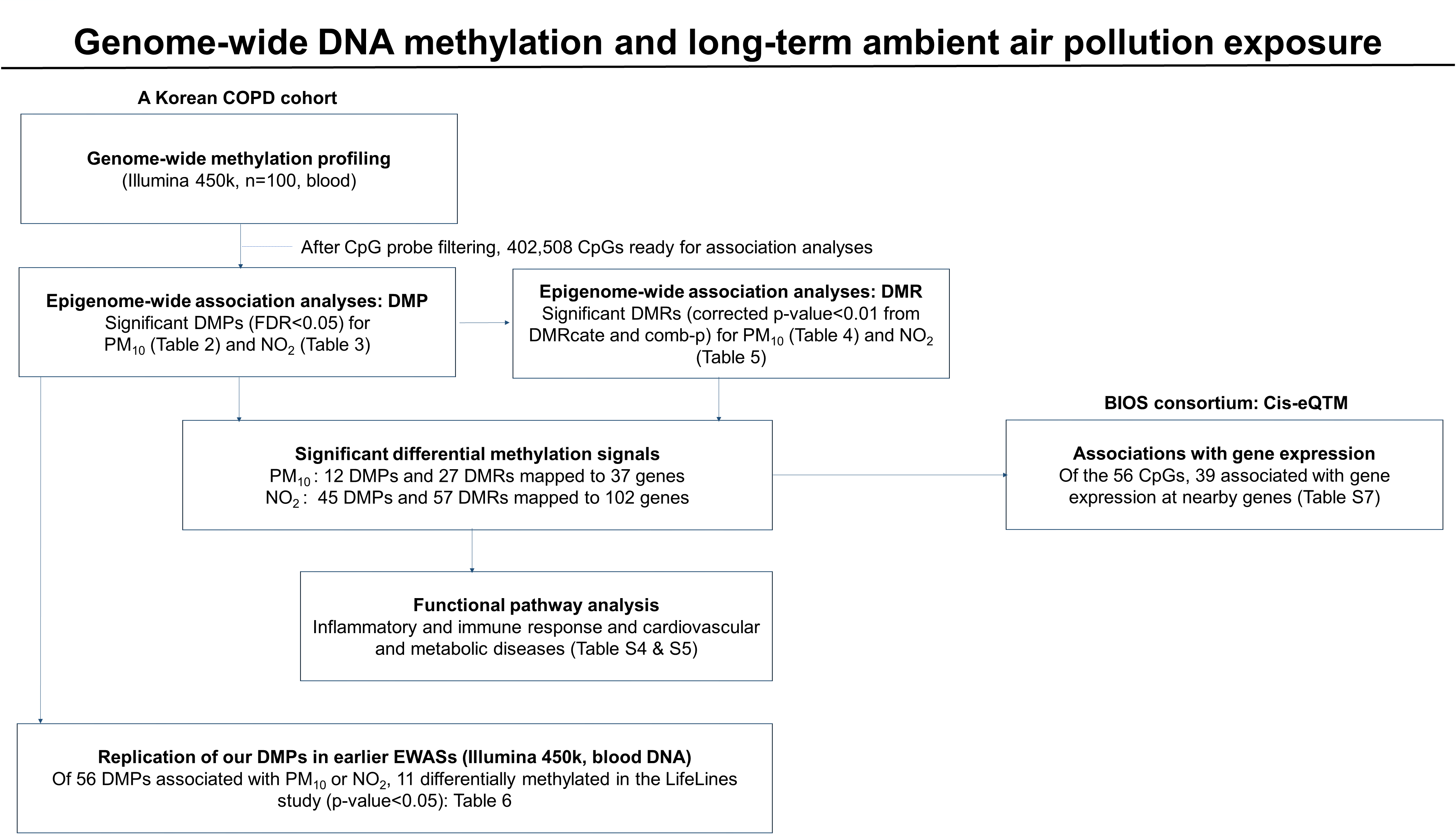


Figure S2. Manhattan and quantile-quantile plots

1. Manhattan plot: EWAS of long-term exposure to PM_10_


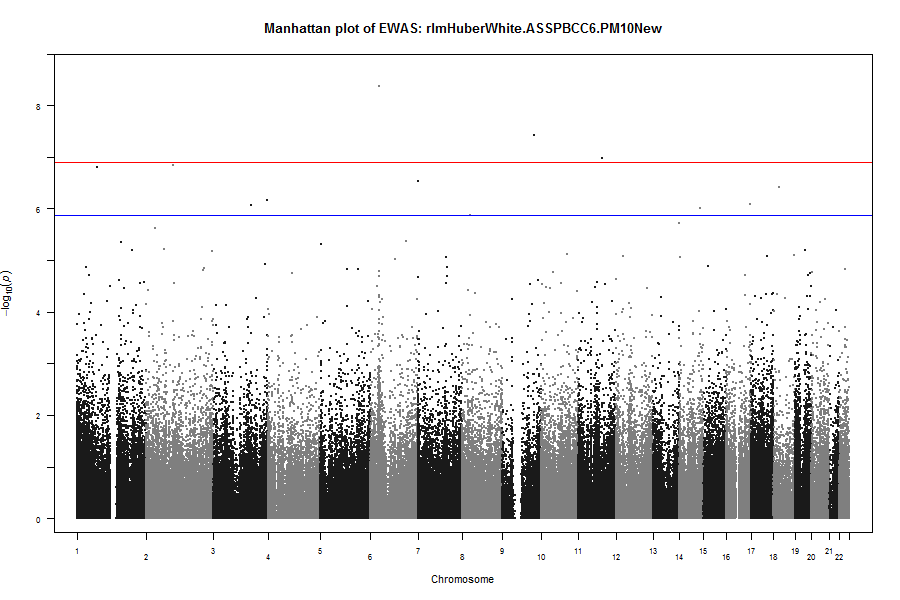


The manhattan plot shows –log_10_P on chromosomal locations. The horizontal lines in red and blue represent thresholds of genome-wide significance of Bonferroni (0.05/402508=1.2E-07) and false-discovery rate (FDR of 0.05), respectively.

1. Quantile-quantile plot: EWAS of long-term exposure to PM_10_


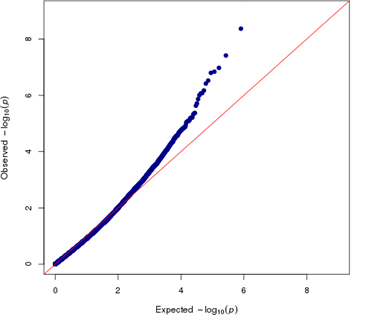


The Q-Q plot shows observed versus expected –log_10_P. Genomic inflation factor (lambda) was 0.83.

1. Manhattan plot: EWAS of long-term exposure to NO_2_


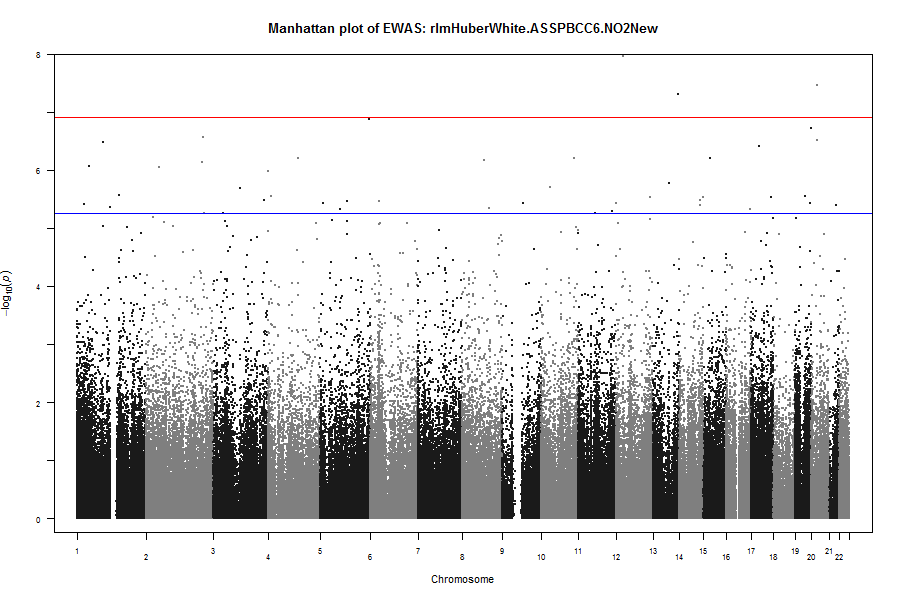


The manhattan plot shows –log_10_P on chromosomal locations. The horizontal lines in red and blue represent thresholds of genome-wide significance of Bonferroni (0.05/402508=1.2E-07) and false-discovery rate (FDR of 0.05), respectively.

1. Quantile-quantile plot: EWAS of long-term exposure to NO_2_


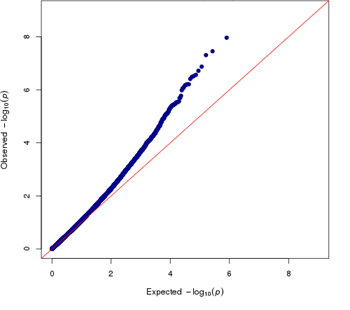


The Q-Q plot shows observed versus expected –log_10_P. Genomic inflation factor (lambda) was 1.07.

Figure S3. Regional visualization of the association of air pollution exposure (PM_10_ and NO_2_) with blood DNA methylation

1. Differential methylation at *TRIM39* in relation to PM_10_ exposure


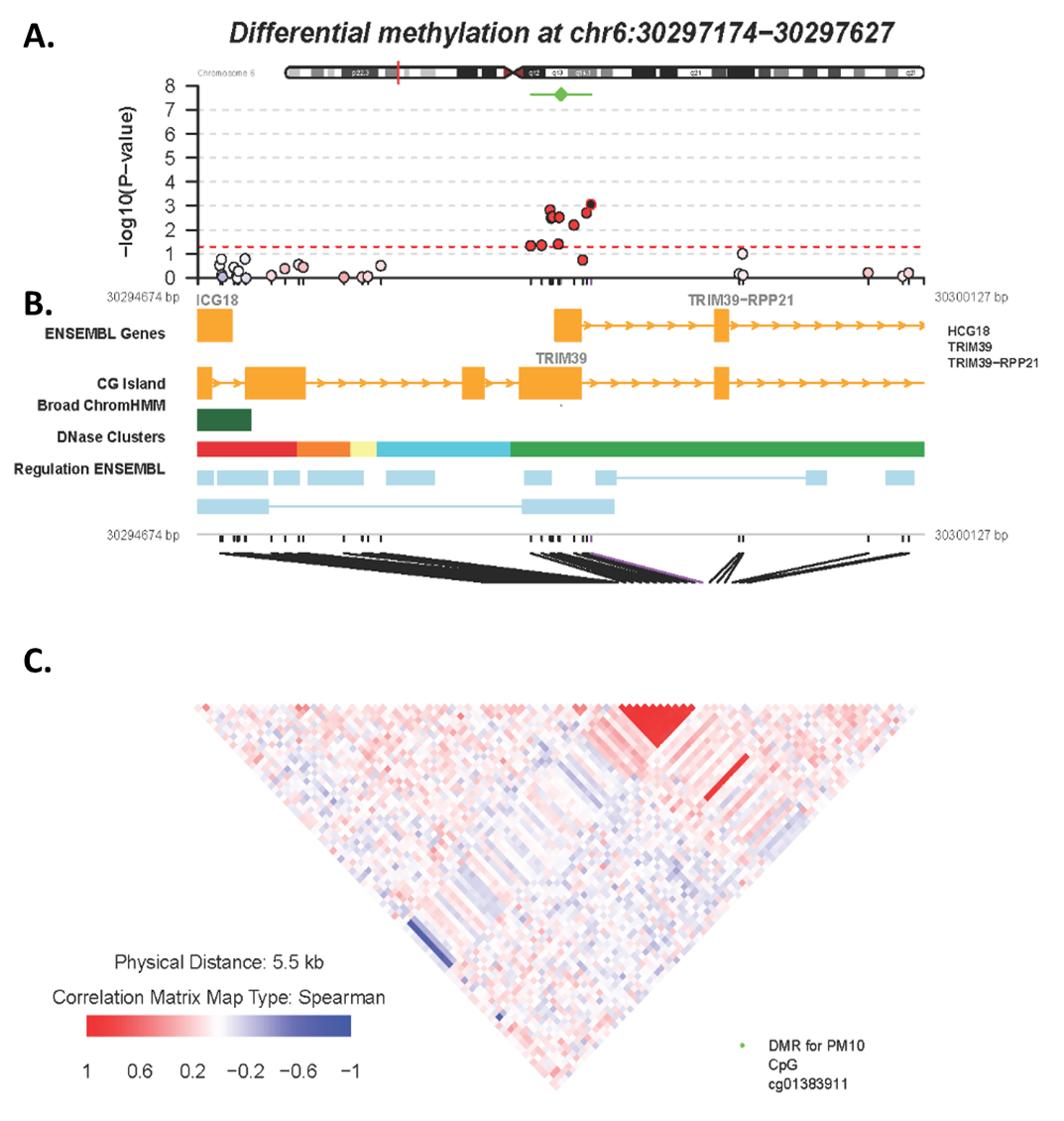


A. Association results of PM_10_ with methylation. Statistical significance for the differential methylation region in green represents FDR from DMRcate. Green horizontal line with diamond shape represents differential methylation region of PM10. Individual CpGs are shown in circles with color indicating pairwise correlation with neighboring CpGs. B. Annotation tracks for the genomic region. Genes, regulatory region of the region from ENSEMBLE, Digital DNaseI Hypersensitivitiy Clusters from ENCODE (DNase Cluster), and Chromatin State Segmentation by HMM from ENCODE/Broad (Broad ChromHMM) are indicated. C: Pairwise correlation of methylation levels at CpGs in the region.

1. Differential methylation at *LTA* in relation to PM_10_ exposure

*
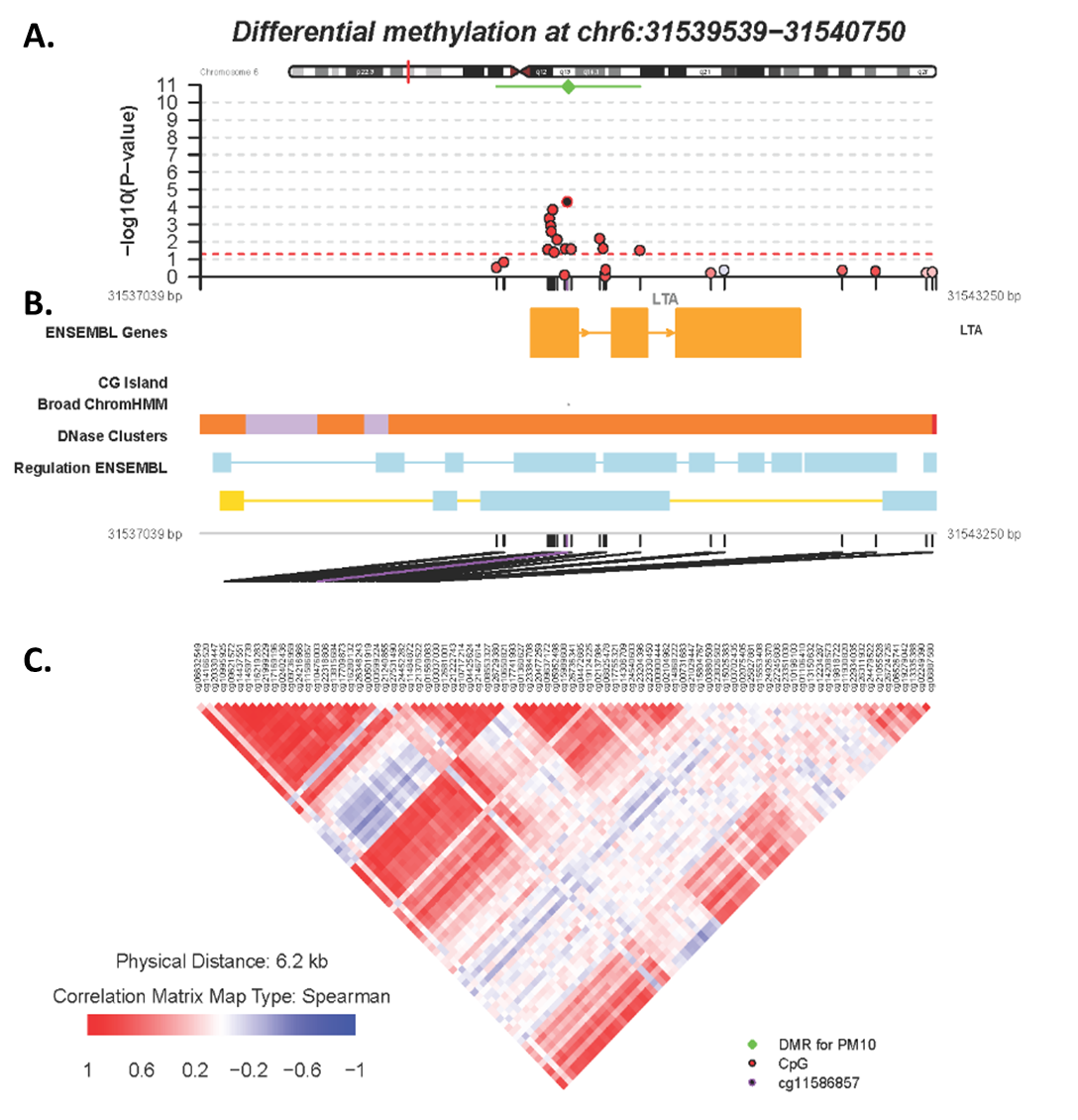
*

A. Association results of PM_10_ with methylation. Statistical significance for the differential methylation region in green represents FDR from DMRcate. Green horizontal line with diamond shape represents differential methylation region of PM10. Individual CpGs are shown in circles with color indicating pairwise correlation with neighboring CpGs. B. Annotation tracks for the genomic region. Genes, regulatory region of the region from ENSEMBLE, Digital DNaseI Hypersensitivitiy Clusters from ENCODE (DNase Cluster), and Chromatin State Segmentation by HMM from ENCODE/Broad (Broad ChromHMM) are indicated. C: Pairwise correlation of methylation levels at CpGs in the region.

1. Differential methylation at *LTA* in relation to NO_2_ exposure


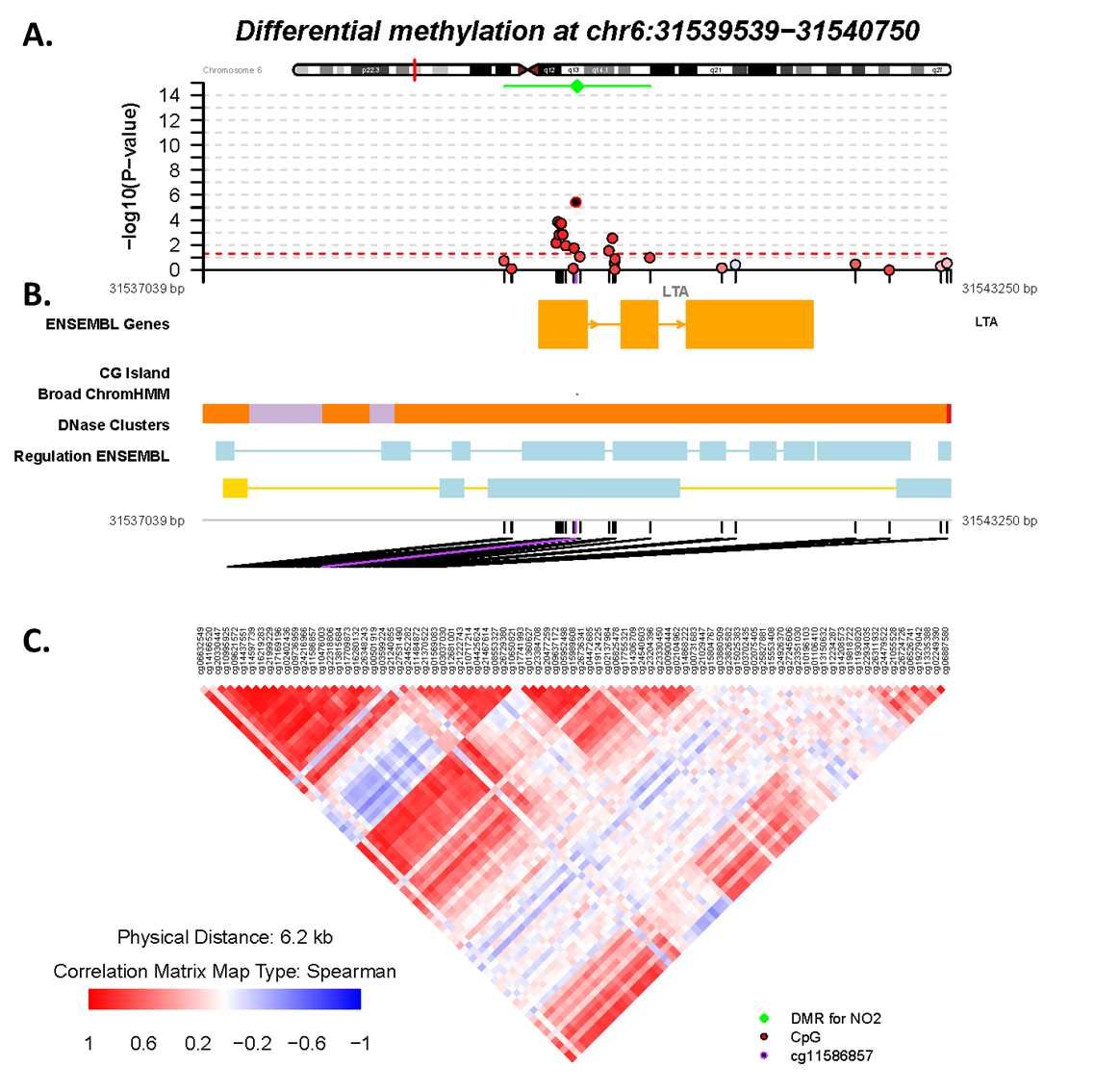


A. Association results of NO_2_ with methylation. Statistical significance for the differential methylation region in green represents FDR from DMRcate. Green horizontal line with diamond shape represents differential methylation region of PM10. Individual CpGs are shown in circles with color indicating pairwise correlation with neighboring CpGs. B. Annotation tracks for the genomic region. Genes, regulatory region of the region from ENSEMBLE, Digital DNaseI Hypersensitivitiy Clusters from ENCODE (DNase Cluster), and Chromatin State Segmentation by HMM from ENCODE/Broad (Broad ChromHMM) are indicated. C: Pairwise correlation of methylation levels at CpGs in the region.

1. Differential methylation at *TRIM39* in relation to NO_2_ exposure


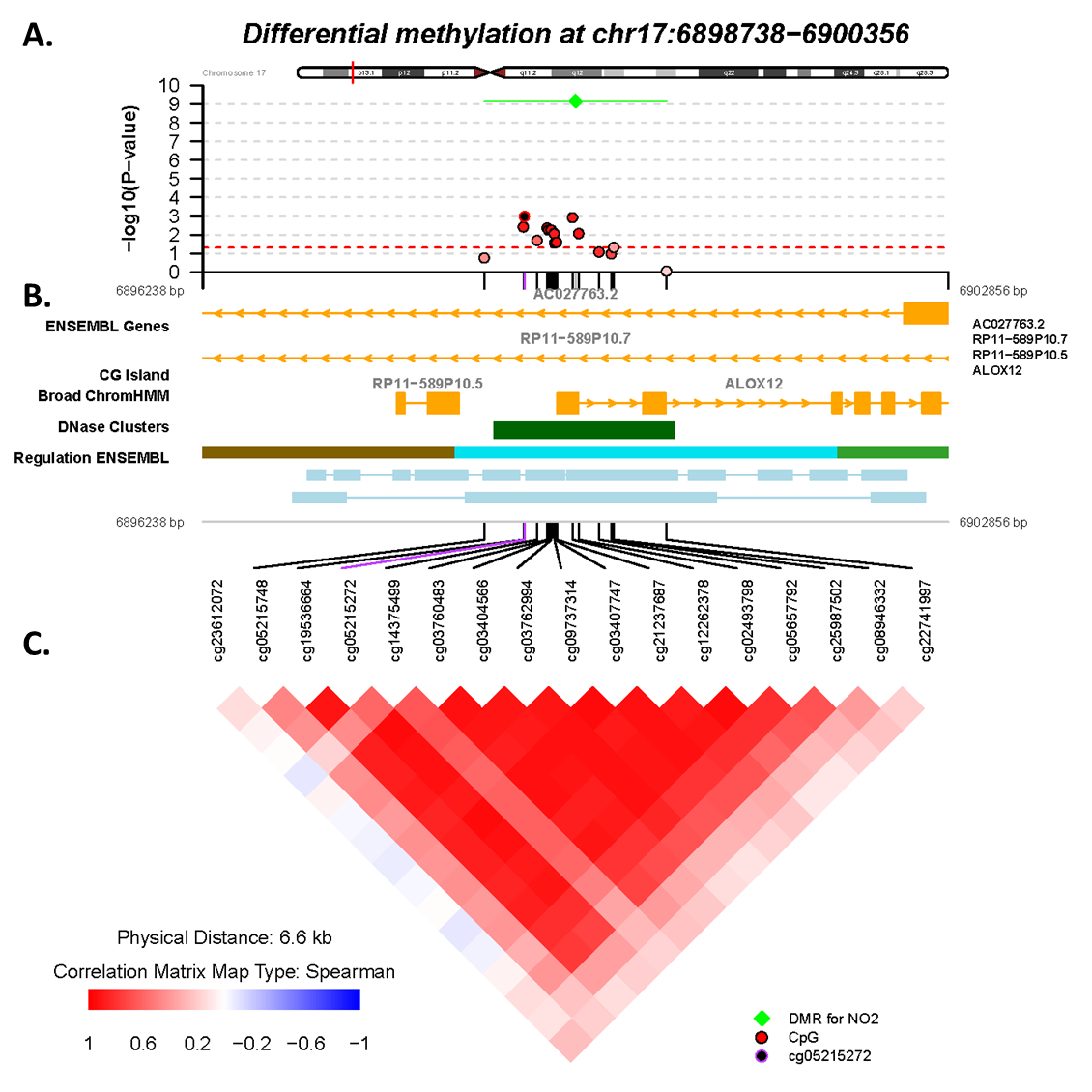


A. Association results of NO_2_ with methylation. Statistical significance for the differential methylation region in green represents FDR from DMRcate. Green horizontal line with diamond shape represents differential methylation region of PM10. Individual CpGs are shown in circles with color indicating pairwise correlation with neighboring CpGs. B. Annotation tracks for the genomic region. Genes, regulatory region of the region from ENSEMBLE, Digital DNaseI Hypersensitivitiy Clusters from ENCODE (DNase Cluster), and Chromatin State Segmentation by HMM from ENCODE/Broad (Broad ChromHMM) are indicated. C: Pairwise correlation of methylation levels at CpGs in the region.

Figure S4. Visualization of pathway analysis results

1. Heatmap showing enrichment of genes (rows) and pathways (columns). Rows and columns are hierarchically clustered using hcluster() function in the R amap package. The gene dendrogram was cut into 8 branches, the pathway category dendrogram was cut into 7 branches. The top color bar indicates in purple (NO_2_) or yellow (PM_10_) whether a given category contained one or more significantly enriched pathway and is shaded based upon the lowest enrichment P-value per category. The left color bar indicates in purple (NO_2_) or yellow (PM_10_) whether each gene was present in the NO_2_ or PM_10_ association results.

1. Genes in six pathways, within the immunology-related category (Cluster 4 in Figure S4. A), related to PM_10_ and/or NO_2_. Each pathway is connected to gene nodes, where gene nodes are colored purple (NO_2_), yellow (PM_10_), or purple/yellow (NO_2_/ PM_10_) to indicate whether a CpG in the gene was associated with NO_2_ and/or PM_10_ exposure.

Figure S5. Tissue- and cell-type specific enrichment pattern in CpGs significantly associated (FDR<0.05) with PM_10_ exposure

1. DNase1 sites (probably transcription factor binding sites) in cell lines for H3K4me1 in Consolidated Roadmap Epigenomics data

Figure S6. Tissue- and cell-type specific enrichment pattern in CpGs significantly associated (FDR<0.05) with NO_2_ exposure

1. DNase1 sites (probably transcription factor binding sites) in cell lines for H3K4me1 in Consolidated Roadmap Epigenomics data

1. DNase1 sites (probably transcription factor binding sites) in cell lines for H3K27me3 in Consolidated Roadmap Epigenomics data

1. DNase1 sites (probably transcription factor binding sites) in cell lines for H3K4me3 in Consolidated Roadmap Epigenomics data

1. DNase1 sites (probably transcription factor binding sites) in cell lines for H3K9me3 in Consolidated Roadmap Epigenomics data
